# Algogenic substances and metabolic status in work-related Trapezius Myalgia: a multivariate explorative study

**DOI:** 10.1186/1471-2474-15-357

**Published:** 2014-10-28

**Authors:** Björn Gerdle, Jesper Kristiansen, Britt Larsson, Bengt Saltin, Karen Søgaard, Gisela Sjøgaard

**Affiliations:** Department of Pain and Rehabilitation Center and Department of Medical and Health Sciences, Linköping University, SE-581 85 Linköping, Sweden; National Research Centre for the Working Environment, Copenhagen, Denmark; CRMC, University of Copenhagen, Copenhagen, Denmark; Institute of Sport Sciences and Clinical Biomechanics, University of Southern Denmark, Campusvej 55, 5230 Odense M, Denmark

**Keywords:** Myalgia, Exercise, Human, Mental stress, Microdialysis, Pain

## Abstract

**Background:**

This study compares the levels of algesic substances between subjects with trapezius myalgia (TM) and healthy controls (CON) and explores the multivariate correlation pattern between these substances, pain, and metabolic status together with relative blood flow changes reported in our previous paper (*Eur J Appl Physiol* 108:657–669, 2010).

**Methods:**

43 female workers with (TM) and 19 females without (CON) trapezius myalgia were – using microdialysis - compared for differences in interstitial concentrations of interleukin-6 (IL-6), bradykinin (BKN), serotonin (5-HT), lactate dehydrogenas (LDH), substance P, and N-terminal propeptide of procollagen type I (PINP) in the trapezius muscle at rest and during repetitive/stressful work. These data were also used in multivariate analyses together with previously presented data (*Eur J Appl Physiol* 108:657–669, 2010): trapezius muscle blood flow, metabolite accumulation, oxygenation, and pain development and sensitivity.

**Results:**

Substance P was significantly elevated in TM (p=0.0068). No significant differences were found in the classical algesic substances (p: 0.432-0.926). The multivariate analysis showed that blood flow related variables, interstitial concentrations of metabolic (pyruvate), and algesic (BKN and K^+^) substances were important for the discrimination of the subjects to one of the two groups (R^2^: 0.19-0.31, p<0.05). Pain intensity was positively associated with levels of 5-HT and K^+^ and negatively associated with oxygenation indicators and IL-6 in TM (R^2^: 0.24, p<0.05). A negative correlation existed in TM between mechanical pain sensitivity of trapezius and BKN and IL-6 (R^2^: 0.26-0.39, p<0.05).

**Conclusion:**

The present study increased understanding alterations in the myalgic muscle. When considering the system-wide aspects, increased concentrations of lactate, pyruvate and K^+^ and decreased oxygenation characterized TM compared to CON. There are three major possible explanations for this finding: the workers with pain had relatively low severity of myalgia, metabolic alterations preceded detectable alterations in levels of algesics, or peripheral sensitization and other muscle alterations existed in TM. Only SP of the investigated algesic substances was elevated in TM. Several of the algesics were of importance for the levels of pain intensity and mechanical pain sensitivity in TM. These results indicate peripheral contribution to maintenance of central nociceptive and pain mechanisms and may be important to consider when designing treatments.

**Electronic supplementary material:**

The online version of this article (doi:10.1186/1471-2474-15-357) contains supplementary material, which is available to authorized users.

## Background

Chronic pain conditions such as neck-shoulder pain including trapezius myalgia have a prevalence in the population of 10-20% and with a higher prevalence in women [1, 2]. The aetiology and pathophysiology of acute myalgia is not fully understood. The diagnoses of chronic myalgia are settled by careful anamnesis and clinical examination relied on a bio-psycho-social model of pain [3]. Acute myalgia usually starts with a feeling of tiredness and stiffness. In an, initially intermittent stage, pain often can be alleviated for short or long periods. Chronic regional myalgia in the shoulder area often gradually becomes more easily triggered and can be spread to include most of the body; chronic wide spread pain. Patients usually report more or less ongoing pain. During clinical examinations, palpation often reveals tender muscles corresponding to the reported painful areas.

Pain is a complex process that involves the interaction of an array of biochemical transmitters and receptors in both the peripheral and central nervous systems. Chronic pain is associated with alterations in the central nervous system (CNS) such as central hyperexcitability, alterations in the pain matrix in the cerebrum and in the descending control of nociception [4–12]. Muscle nociception is activated by stimulation of free nerve endings of group III (Aδ) and IV afferent (C) fibres. Nociceptors respond to single or combinations of noxious stimuli and their sensitivity can be increased by endogenous substances [13–15]. So the question arises whether muscle alterations with respect to metabolics and algesics are present in chronic myalgia and contribute to maintenance of the central alterations mentioned above.

The microdialysis (MD) technique offers an in vivo method to study nociceptive and metabolic mechanisms in chronic myalgia [16]. MD allows for continuous sampling of compounds in the muscle interstitial space (i.e., the extra cellular fluid), where nociceptor free nerve endings terminate and in close proximity to the muscle fibers providing accurate information on local biochemical changes before such compounds are diluted and cleared by the circulatory system. Hence, the extracellular matrix plays a key role in the functions of the nociceptors [17]. We have investigated the interstitial milieu of the myalgic trapezius muscle as part of a *chronic* regional neck-shoulder pain condition in women with respect to three degrees of severity [18–20]. Increases in algesics e.g., serotonin (5-HT), glutamate, and kallidin in chronic trapezius myalgia and other myalgic muscles have been reported [18–24]. Increases in interstitial concentrations of lactate and pyruvate have also been found in these cohorts of trapezius myalgia [19, 20]. These results were recently confirmed in female workers with trapezius myalgia active in the labour market and with *relatively low severity*[25].

Hence both the briefly referred studies and other studies indicate that a number of substances can be released and altered in the milieus of the nociceptors not only in acute nociception (“the inflammatory soup”) but also in chronic myalgias [26]. There is growing consensus that a panel of multiple biomarkers will perform better than a single biomarker in the understanding of activated nociceptive and pain mechanisms. Traditional statistical methods can quantify level changes of individual substances [27] but assume variable independence and disregard interrelationships between variables [28]. The development of *Omics* methods meaning large-scale –data analysis for characterization and quantification of pools of biological molecules has promoted the development of statistical methods capable of handling a number of intercorrelated substances. In order to handle situations when traditional classical statistical assumptions not are met or appropriate Multivariate Data Analysis (MVDA) has been developed; e.g., advanced principal component analyses and Partial Least Squares regressions. Hence, these methods represent a complementary approach to the traditional statistical methods for better understanding of the complex biochemical alterations that may occur in chronic musculoskeletal pain; these methods have been applied in other MD studies of chronic myalgia [23, 29].

The present study focuses on mainly algesic substances - possible to analyse from dialysate and investigated in earlier MD studies - and how these substances interact with earlier reported metabolic and blood flow alterations in the same subjects [25]. Thus, this study has three main aims:

1. to compare the concentrations of glutamate, bradykinin (BKN), 5-HT, Lactate dehydrogenase (LDH), interleukin-6 (IL-6), substance P (SP), and N-terminal propeptide of procollagen type I (PINP) between trapezius myalgia subjects (TM) and healthy controls (CON);
2. to identify substances that in the multivariate context best separate CON from TM;
3. to investigate the multivariate associations between aspects of pain (intensity and sensitivity), algesics, and metabolic status together with oxygenation and blood flow changes reported in our previous paper in the two groups of subjects [25].

## Methods

### Subjects

A case–control study was performed and female workers were recruited from seven workplaces, which were characterized by typically monotonous and repetitive work tasks; for a full description of the recruitment process including a flow-chart, see our previous article [25]. Two groups of subjects were recruited: 1) subjects with trapezius myalgia (TM) and 2) healthy subjects (CON).

The following criteria had to be fulfilled for inclusion in the TM group: (1) pain or discomfort in the neck/shoulder region for more than 30 days during the previous year; (2) not more than 30 days of pain or discomfort in no more than three out of eight major body regions (neck/shoulder, low back, and left or right arm/ hand, hip, knee/foot) – this criterion was used to exclude widespread musculoskeletal diseases; (3) the pain or discomfort should be at least *quite a lot* on an ordinal 5-step scale ranging from *a little* to *very much*; (4) the pain or discomfort should be frequent (at least once a week); and (5) the intensity of the pain or discomfort should be at least 2 on a scale from 0 to 9, where 0 is *no pain* and 9 is the *worst imaginable pain*[30]. 6) A clinical standardized examination for the confirmation of the diagnosis trapezius myalgia [31, 32]; the main criteria for a positive clinical diagnosis were (a) pain in the neck area, (b) tightness of the trapezius muscle, and (c) palpable tenderness in the trapezius muscle. According to the inclusion and exclusion criteria, TM had chronic pain of relatively low severity compared to patients no longer on the labour market. For inclusion in CON, the following criteria had to be fulfilled: (1) pain or discomfort for less than eight days during the previous year in the neck/shoulder region; and (2) no more than three body regions with more than 30 days of pain or discomfort, and negative replies were requested regarding question (3) to (5). Additionally, none of the participants should suffer from serious conditions such as previous trauma or injuries, life threatening diseases, cardiovascular diseases, or arthritis in the neck and shoulder. In order to describe the work situation of all subjects answered a brief questionnaire that included questions regarding the job and the work ability (i.e., work ability index) [33, 34]. In total, 43 TM and 19 CON participated in this study and successful MD data were obtained from all of the TM and 17 of the CON participants. As earlier reported age, height, and weight showed no group differences (Table 1) [25]. As also reported earlier TM reported significantly higher pain intensity at rest, a significantly lower work ability index but without significant differences in sick leave (Table 1). The majority of TM and CON reported that they seldom (never, hardly never or 2–3 times recent month) used medication (71.4% vs. 90%) and daily use of medication (once or several times a day) were reported by 23.7% in TM and 10% in CON. Two thirds of the medication used was analgesics.

**Table 1:** **Background data together with current pain intensity, work ability index and sick leave**

Group variables	CON Mean ± SD	TM Mean ± SD	Statistics (p-value)
Age (years)	44 ± 9.1	44 ± 9.8	ns
Height (m)	1.68 ± 0.06	1.65 ± 0.06	ns
Weight (kg)	70 ± 10.6	72 ± 15.0	ns
Pain intensity (VAS, mm)	2.7 ± 3.5	27 ± 22	<0.001
Work ability index	39.1 ± 2.0	35.9 ± 3.5	0.01
Sick leave last year (days)	5 ± 8	8 ± 16	ns

CON denotes healthy controls) and TM denotes subjects with trapezius myalgia; these data has been reported earlier [25]. Furthest to the right is shown the statistical evaluation (p-values); ns denotes no significant group difference.

All subjects gave written informed consent, which conformed to The Declaration of Helsinki and was approved by the Capital Region of Denmark ethical committee (KF 01-138/04). The study qualified for registration in the International Standard Randomized Controlled Trial Number Register ISRCTN87055459, registration date: 14 March 2014. The cases of the present study form the baseline population of a previously reported randomized controlled trial (RCT) [35]. The present case control study is included in the clinical trial registration. CONSORT guideline related information and CONSORT flowchart is found in the article reporting the RCT [35].

### Procedures

Pressure pain thresholds (PPT) – i.e., mechanical pain sensitivity - were determined several days before the MD session. The participants were asked not to use any medications except for paracetamol preparations three days before the MD session day and were instructed not to perform any shoulder or neck-training exercises for 48 h before the session, except for ordinary daily work and/or leisure activities. The participants reported to the laboratory in the morning. They finished breakfast one to two hours before the start of MD and had standardized light meals at frequently set time-points throughout the experiment to maintain blood glucose and digestion as constant as possible. The MD catheters were inserted into the trapezius muscle of the most painful side in case of side differences for the TM group. For 30% of the TM group (13 out of 43), the non-dominant side was most affected and therefore 30% of the CON group had the MD catheter inserted into the non-dominant side (5 out of 17); in all, 10% were left-hand dominant. Then the participants rested for 120 min to allow the tissue to recover from possible changes in the interstitial environment induced by the catheter insertion. During the resting period, near-infrared spectroscopy (NIRS) sensors were mounted above the descending part of the trapezius muscle above the MD permeable catheter part. The resting period was followed by a 40-min repetitive low-force exercise period performed unilaterally on a pegboard (PEG) using the hand on the same side the MD catheter had been inserted in the trapezius muscle. Following this exercise, the participants rested for 120 min (recovery). The final 20 min of the recovery was used as a baseline for a stressful task: The STROOP test (STR) for 10 min with a subsequent 30-min recovery period [25].

### Pressure pain thresholds (PPT)

PPTs were measured bilaterally over trapezius and tibialis anterior muscles using an algometer (Algometer Type 2, Somedic, Hörby, Sweden) with a diameter of the contact area of 10 mm and a pressure applied perpendicular to the skin at a speed of 30 kPa/s; for details see [36].

### Pain intensity (VAS)

Pain intensity of the shoulder region was assessed throughout the MD session with a 100-mm visual analogue scale (VAS), ranging from 0 mm (*no pain*) to 100 mm (*worst possible pain*). The shoulder region was defined as the area covered by m. trapezius’ descending part, m. supraspinatus and m. infraspinatus. VAS was rated on the experimental working-day before and immediately after insertion of the MD catheter and every 60 min during rest; these values were not significantly different from the recording at 160 min synchronised to the MD sampling (see below) and taken as baseline before the PEG. During the PEG task, VAS was rated every 5 min (i.e., eight times during the 40 min PEG) and then at 10, 60, and 120 min post exercise. VAS was also rated immediately before (320 MD time) and after the STR task and in the following recovery period every 10 min.

### Microdialysis (MD)

**Figure 1:**
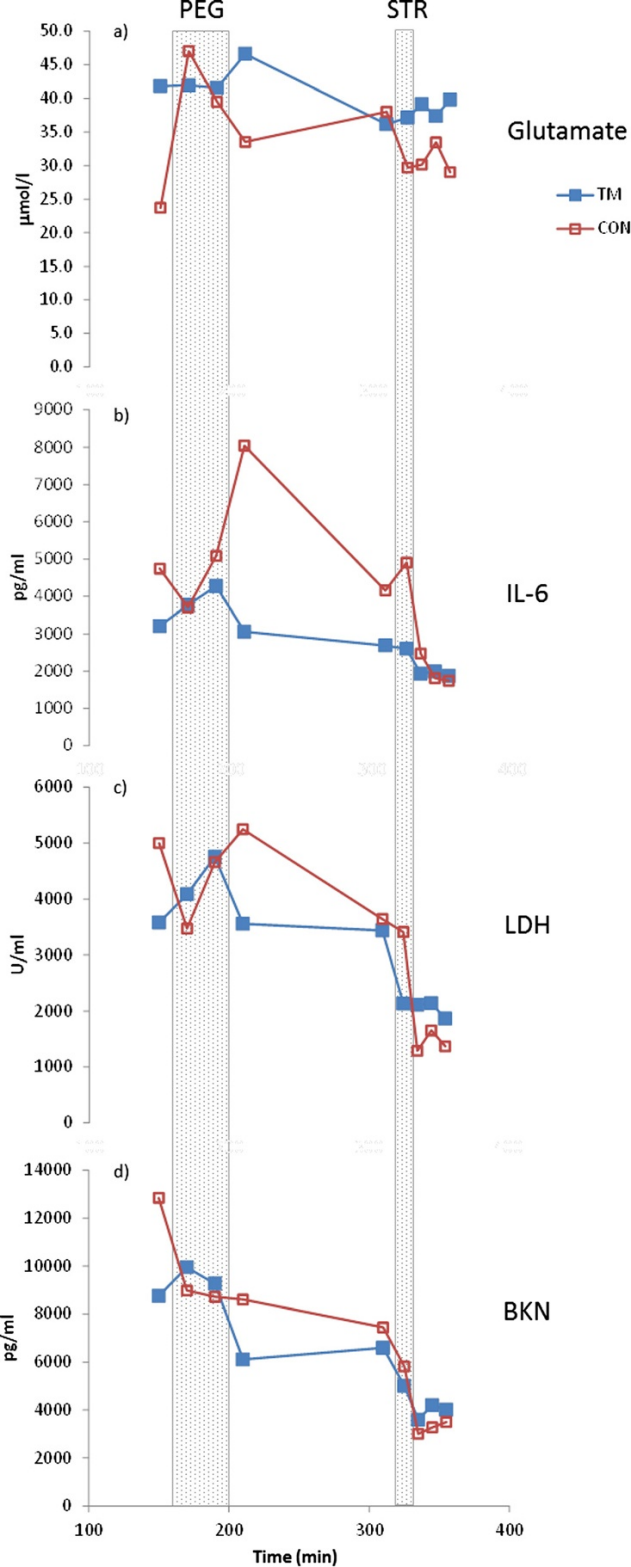
**Mean interstitial concentrations of glutamate (panel a), interleukin 6 (IL-6; panel b), lactate dehydrogenase (LDH; panel c), and bradykinin (BKN; panel d) throughout the microdialysis experiment in women with trapezius myalgia (TM) and in healthy controls (CON).** X-axis gives min after insertion of the catheters and samples are plotted at midpoint of the sampling period. PEG = repetitive low-force exercise performed unilaterally on a pegboard during 40 min (160–200 min after catheter insertions), STR = STROOP test during 10 min (320–330 min after catheter insertions). TM is denoted by filled blue squares and CON is denoted with unfilled red squares.

MD was performed as previously described [25]. In summary, two custom-made MD catheters (membrane length 30 mm, molecular cut-off: 5 kDa and 3000 kDa, inter-catheter distance approximately 2 cm) [37] were inserted in the trapezius muscle parallel to the muscle fibres. The MD catheters were perfused by a high precision syringe pump (CMA 100; Carnegie Medicine, Solna, Sweden) at a rate of 5 μl min^−1^ with a Ringer acetate solution (Pharmacia & Upjohn, Copenhagen, Denmark) containing 3 mM glucose and 0.5 mM lactate to minimise the risk of draining the interstitial space [38]. 1.0 M [^14^C]-lactate (specific activity: 2.22 GBq mmol^−1^; Amersham, Bucks, UK) was added to the perfusate to determine the in vivo relative recovery (RR) of lactate, pyruvate, and glucose (each approximately similar in molecular size and weight) using the internal reference method [25, 39]. Furthermore, nutritive trapezius muscle blood flow was estimated by the MD ethanol technique using ^3^H_2_O instead of ethanol [40, 41]. The ratio of ^3^H_2_O in the dialysate and the perfusate (the outflow-to-inflow ratio) varies inversely with the local blood flow in the tissue [40, 41]. Microdialysates were collected continuously into MD vials that were changed every 20 min starting at t = 20 min until t = 320 min, and thereafter every 10 min up to t = 360 min (Figure 1). The samples collected from t = 20-100 min were not used in this study. As Dialysate samples were collected and the samples were immediately frozen and stored at −80°C until analyses were performed. The sample volume collected at each time point was relatively small (50–100 μl), not all biochemical components could be determined in all samples. Thus, dialysate from t = 120 and t = 140 min were pooled and used to determine PINP, serotonin, and substance P (see below). Glutamate, lactate, pyruvate, glucose, potassium, LDH, BKN, as well as local blood flow were determined in samples collected at 160 min (baseline before PEG), 180 min (PEG), 200 and 220 min (recovery after PEG), 320 min (baseline before STR), 330 min (STR), and 340, 350, and 360 min (recovery after STR). The average number of dialysate samples possible to use for the determination of concentrations of algesics was 92% (except SP; see below). Because the measured concentrations refer to the average interstitial concentrations in the period during which the dialysate was collected, the time assigned to each sample was the time midway in the collection period. Lactate, pyruvate, glucose, K^+^, 5-HT, SP, and local muscle blood flow was determined in microdialysate collected using a 5 kDa catheter, and LDH, IL-6, PINP and BKN were determined in samples using a 3000 kDa catheter.

Concentration of muscle interstitial glutamate ([glutamate]) was measured in a CMA 600 Microdialysis Analyzer (CMA Microdialysis, Stockholm, Sweden). The CMA 600 detection interval is 1.0 -150 μmol l^−1^ for glutamate. Concentrations below the level of detection were replaced with a value corresponding to the lower level of detection and divided by two [24]. Muscle interstitial LDH activity ([LDH]) was measured by a Cytotoxicity Detection Kit (Roche Molecular Biochemicals, Roche Diagnostics, Mannheim, Germany), which has a level of detection of 21.5 mU/ml. Muscle interstitial IL-6 concentration ([IL-6]) was measured using a high-sensitivity Quantikinew assay (R&D systems, Minneapolis, MN, USA). In order to meet the minimum sample volume requirements of the IL-6 assay, dialysate samples were diluted 100-fold with calibrator diluent. The lowest calibration standard was used as the level of detection. Hence, the detection level for IL-6 in the dialysate was 15.6 pg/ml when adjusting for the dilution factor. Accuracy of the assay was checked by spiking buffer and a low sample in duplicate with interleukin-6 international standard (NIBSC 89/548) [42]. Muscle interstitial BKN ([BKN]) was measured by a radioimmunoassay (Peninsula Laboratories, Inc., Bachem AG, Bubendorf, Switzerland). The limit of detection in the dialysate was 242 pg/ml. Muscle interstitial 5-HT concentrations ([5-HT]) were measured with an enzyme immunoassay (Immunotech, Marseille, France) with a detection level of 0.28 ng/ml. In order to make a meaningful statistical comparison of 5-HT levels between groups, 5-HT results below the level of detection was recorded as half of the detection level [43]. Muscle interstitial concentration of SP ([SP]) was determined with an enzyme immunoassay (Assay Designs, Enzo Life Sciences, Farmingdale, NY, USA), the limit of detection in the dialysate was 2.0 pg/ml. Muscle interstitial collagen synthesis was determined as the concentration of PINP ([PINP]). [PINP] were determined using a sandwich ELISA technique with a detection limit of 0.071 Ng/ml [44].

### Previously described methods and reported results

Details concerning the methodology of Near-infrared spectroscopy (NIRS) and the methods for determination of concentration of metabolites have been described in our previous article from this study and are not repeated here [25]. The results concerning NIRS data, pain intensity, muscle interstitial lactate, pyruvate, and glucose concentrations ([lactate], [pyruvate], and [glucose], respectively), muscle dialysate potassium concentration ([K^+^]) and blood flow (i.e., the ratio of ^3^H_2_O in the dialysate and the perfusate - the outflow-to-inflow ratio - varies inversely with the local blood flow in the tissue flow) with respect to group and time as well as PPT data have been described in detail in our previous article [25] and are summarized in Additional files 1 and 2. Note that this data from our previous article *only* is used in the multivariate analyses of the present study.

### Statistics

Data are presented as mean ± one standard deviation (±1SD) unless otherwise specified. SPSS (Version 11.0, SPSS Inc., Chicago) was used for the classical statistical analyses. Analysis of variance (ANOVA) for repeated measures using a first order autoregressive covariance structure model was used to test for time and group effect during the two separate exercise periods and their respective recovery periods; for details [25].

For investigating the multivariate correlation patterns between the interstitial concentrations of different metabolic and algesic substances, pain intensity, relative blood flow changes, etc., *Principal component analysis* (PCA) and *Partial least squares* or *projection to latent structures* (PLS-OPLS/O2PLS) were applied using SIMCA-P + [45]. These methods are increasingly applied in situations with large data sets and with low subject-to-variables ratios [46, 47].

*Principal component analysis* (PCA) can be viewed as a multivariate correlation analyses. Variables loading upon the same component (p) are positively correlated, and variables with high loadings but with different signs are negatively correlated. Variables with high absolute loadings with respect to the component under consideration were considered significant [45]. The obtained significant components are per definition not correlated. R^2^ describes the *goodness of fit* and Q^2^ describes the *goodness of prediction*[45]. Outliers were identified using the two methods available in SIMCA-P+. Two multivariate outliers were identified– one from CON and one from TM – and excluded in the multivariate analyses.

PLS-OPLS/O2PLS was used for the multivariate regression analysis of pain intensities, pressure pain thresholds, and group membership (CON or TM; coded 0 and 1, respectively) using the interstitial concentrations of the different compounds and other variables as regressors [45]. The VIP variable (variable influence on projection) indicates the relative relevance of each X-variable. VIP ≥ 1.0 was considered significant. Coefficients were used to note the direction of the relationship (positive or negative). VIP values are reported in descending order and the sign of the coefficient is also given. PLS regressions (except for group membership) were performed both for all subjects taken together and in the two groups (TM and CON) separately. This strategy gives the opportunity to detect substances involved in sensitization in TM. A probability of ≤ 0.05 (two-tailed) was accepted as the criterion for significance in all statistical tests.

## Results

### Interstitial concentrations of algesic substances

**Figure 2:**
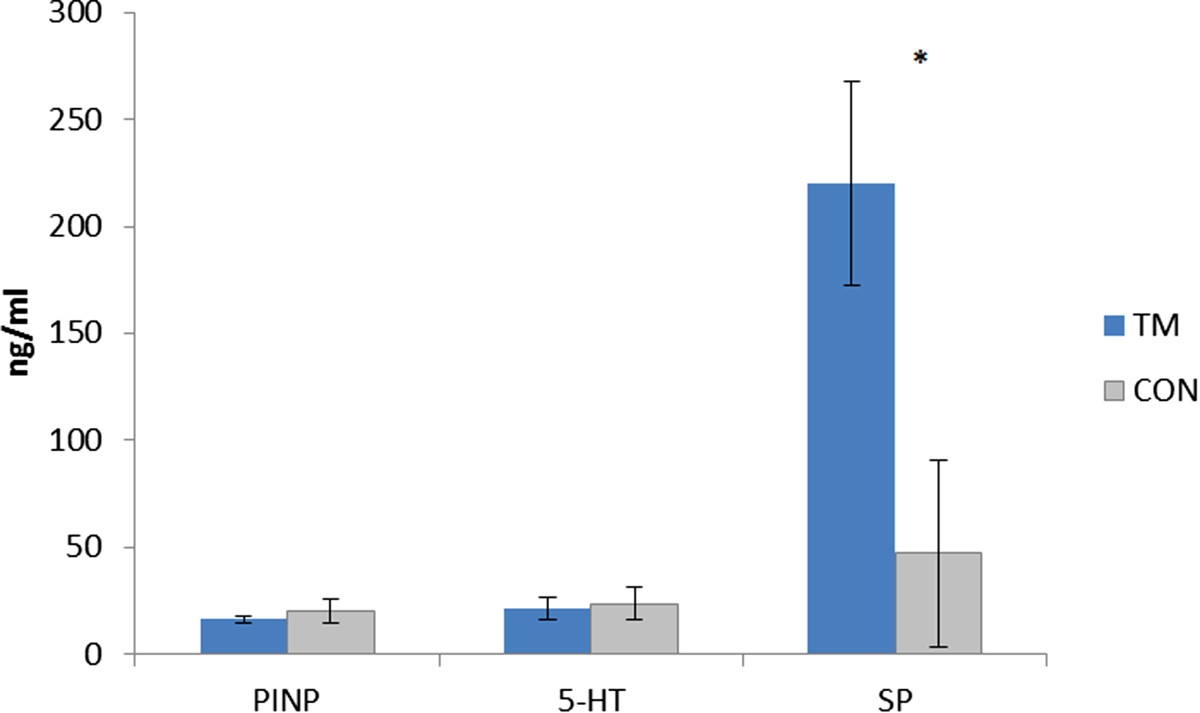
**Mean concentrations (± standard error of the mean; SEM) of N-terminal propeptide of procollagen type I (PINP), serotonin (5-HT) and substance P (SP) in women with trapezius myalgia (TM; blue bars) and in healthy controls (CON; grey bars) based on pooled dialysate (from t = 120 min and t = 140 min; see text for details).** Please note that the number of subjects in CON (n = 6) for SP was low due to lack of dialysate when this analysis was performed.

No group differences were found for the concentrations of glutamate, IL-6, LDH and BKN (Table 2). All substances showed time effects (Table 2 and Figure 1). Similar results and without group differences were obtained when the PEG and STR parts were analysed separately for each of these substances (data not shown). No group differences in [5-HT] (CON: 23.8 ± 29.1 vs. TM: 21.6 ± 33.2; p = 0.432) and [PINP] (CON: 20.1 ± 21.5 vs. TM: 16.4 ± 12.1; p = 0.489) were found (Figure 2). TM had significantly higher [SP] than CON (TM: 220.0 ± 272.5 vs. CON: 47.3 ± 106.5; p = 0.0068) (Figure 2). Please note that the number of subjects in CON (n = 6) was low due to lack of dialysate when the analysis of this substance was performed.

**Table 2:** **The statistical evaluations of the concentrations of glutamate, IL-6, LDH and BKN**

Substance	Group (p-value)	Time (p-value)	Interaction (p-value)
[glutamate]	0.524	<0.001*	na
[IL-6]	0.679	<0.001*	na
[LDH]	0.879	<0.01*	na
[BKN]	0.926	<0.001*	na

P-values for group (CON vs. TM) and time effects are shown. Interaction terms were only calculated when both time and group were significant; na denotes not applicable. *denotes significance.

### Multivariate analyses

Additional file 1 and Additional file 2 summarize the results presented in our previous study for all subjects taken together concerning pain aspects, the interstitial concentrations of metabolites, blood flow (i.e., outflow/inflow ratio), and NIRS data [25]. Note that these data are used in the present study *only* in the multivariate analyses presented below.

### Intercorrelations between biochemical substances, nirs data, and blood flow in the two groups

The multivariate correlation patterns between algesics and metabolites, NIRS data, and blood flow were investigated using PCA in order to investigate if different patterns existed in TM and CON.

The PCA *in CON* identified three significant components (R^2^ = 0.52). According to the *first component* (p1; R^2^ = 0.25), positive inter-correlations existed between [lactate] (six of nine time points), [glutamate] (two out of nine time points), [K^+^] (four of nine time points), and [BKN] (two time points). These variables correlated negatively with blood flow (at all nine time points) and [PINP]. The *second component* (p2; R^2^ = 0.15) mainly showed positive intercorrelations between [glucose] (five of nine time points) and deoxy-haemoglobin (HHb; three of six time points). These variables were negatively inter-correlated with [LDH] (five of nine time points). According to the *third component* (p3; R^2^ = 0.11), oxyhaemoglobin (OHb), HHb, and total haemoglobin (THb) at the two recovery time points inter-correlated positively mainly with [IL-6] (three of nine time points). These variables correlated negatively with blood flow (five of nine time points), and with [pyruvate], [glutamate] and [BKN] (two to three time points for each substance).

The PCA *in TM* identified nine significant components (R^2^ = 0.71) and the three most important components (p1-p3) explained 36% of the variation (R^2^ = 0.36). According to the *first component* (p1; R^2^ = 0.16), positive inter-correlations existed mainly between [lactate], [pyruvate], and [glutamate] (at all nine time points for the three substances), with [BKN] (four time points) and with [5-HT] and [SP]. The *second component* (p2; R^2^ = 0.11) mainly showed positive inter-correlations between [LDH] (eight of nine time points) and blood flow (all nine time points). According to the *third significant component* (p3; R^2^ = 0.09), [IL-6] (six of nine time points) correlated positively with blood flow (seven of nine time points) and HHb (two of six time points). These variables correlated negatively with the concentrations of [SP] and [lactate] (three of nine time points).

### Regression of group membership

Group membership – TM versus CON - was regressed using the relevant variables (biochemical substances, NIRS data, and blood flow) at baseline. This significant regression (R^2^ = 0.19) identified that TM membership compared to CON membership was associated with low blood flow (VIP = 2.33(+)), low [BKN] (VIP = 1.30(−)), high [pyruvate] (VIP = 1.20(+)), low OHb (VIP = 1.03(−)), and high [K^+^] (VIP = 1.01(+)).

A PLS regression using all available time points was also made (R^2^ = 0.31) (Table 3). The blood flow variables at different time points were important for predicting group membership, but there were also several other important regressors: [K^+^] at four time points, [pyruvate] at five time points, [IL-6] at two time points, [lactate] at three time points, and [glutamate] at two time points.

**Table 3:** **PLS regression of group membership using the intramuscular variables as regressors (R** ^**2**^ **= 0.31)**

***Variable***	***Time point***	***VIP***	***Sign of coeff***
**[BKN]**	**150 min**	1.43	-
**Blood flow**	**150 min**	2.57	+
	**170 min**	2.44	+
	**190 min**	2.28	+
	**210 min**	2.35	+
	**310 min**	1.83	+
	**325 min**	1.02	+
	**335 min**	1.87	+
	**345 min**	1.60	+
	**355 min**	1.93	+
**HHB**	**Recov1b**	1.20	-
**OHb**	**21-40 min**	1.44	-
	**PEG-0-20 min**	1.13	-
	**recov2**	1.02	-
	**STR**	1.04	-
**[** **Lactate** **]**	**150 min**	1.02	+
	**170 min**	1.05	+
	**190 min**	1.21	+
	**210 min**	1.20	+
	**325 min**	1.02	+
	**325 min**	1.39	+
	**335 min**	1.02	+
**[** **Pyruvate** **]**	**150 min**	1.33	+
	**170 min**	1.30	+
	**190 min**	1.78	+
	**210 min**	1.14	+
	**325 min**	1.41	+
**[** **Glucose** **]**	**310 min**	1.15	+
**[** **Glutamate** **]**	**150 min**	1.06	+
	**170 min**	1.11	-
**[** **K** ^**+**^ **]**	**150 min**	1.11	+
	**190 min**	1.83	-
	**325 min**	1.59	+
	**345 min**	1.30	+
**[** **IL-6** **]**	**210 min**	1.71	-
	**310 min**	1.35	-
**[LDH]**	**335 min**	1.07	+

[**substance**] denotes concentration of the substance. BKN = Bradykinin, HHB = deoxy-haemoglobin, OHB = oxyhaemoglobin, K^+^ = potassium, IL-6 = interleukin 6, LDH = Lactate dehydrogenase, PEG = repetitive low-force exercise performed unilaterally on a pegboard, STR = STROOP test.

Trapezius myalgia (TM) was denoted 1 and healthy controls (CON) was denoted 0. VIP values and sign of coefficient (coeff) are given. Note that the variables are grouped and sorted within each variable with respect to the time points. A positive coefficient indicates that TM has high values on this variable compared to CON and vice versa. Note the inverse construction of blood flow.

### Regressions of pain intensity in TM^a^

Pain intensity at baseline correlated positively (R^2^ = 0.24) with [5-HT] (VIP = 1.53(+)) and [K^+^] (VIP = 1.50(+)), and negatively with THb (VIP = 1.62(−)), HHb (VIP = 1.41(−)), OHb (VIP = 1.23(−)), and [IL-6] (VIP = 1.07(−)).

It was not possible to regress pain intensity throughout the experiment (several Y-variables) in TM using biochemical substances, NIRS data, and blood flow.

### Regressions of mechanical pain sensitivity (PPT)

It was possible *in TM* to regress PPT of trapezius using baseline data (R^2^ = 0.26); a low PPT was associated with high [BKN] (VIP = 2.18(−)), [IL-6] (VIP = 2.14(−)), THb (VIP = 1.10(−)), and high blood flow (VIP = 1.02(−); note the inverse construction of this variable). Metabolic substances were not significant in this regression.

When regressing PPT of trapezius *in TM* using all data (R^2^ = 0.39) (Table 4) [IL-6] at six time points, [BKN] at six time points, [pyruvate] at three time points, [K^+^] at four time points, and [glucose] were the most important regressors.

**Table 4:** **Regression of pressure pain threshold of trapezius in TM using the intramuscular variables (R** ^**2**^ **= 0.39)**

***Variable***	***Time point***	***VIP***	***Sign of coeff***
**Blood flow**	**150 min**	1.14	-
	**170 min**	1.07	-
	**190 min**	1.05	-
	**210 min**	1.40	-
	**335 min**	1.18	-
	**345 min**	1.56	-
**HHB**	**21-40 min**	1.09	-
	**recov1**	1.47	-
	**recov2**	1.36	-
	**STR**	1.03	+
**OHB**	**recov1**	1.27	-
	**recov2**	1.35	-
**THB**	**21-40 min**	1.01	-
	**PEG-0-20 min**	1.17	-
	**recov1**	1.55	-
	**recov2**	1.63	-
**[Lactate]**	**170 min**	1.27	-
	**210 min**	1.06	-
	**335 min**	1.11	-
**[Pyruvate]**	**170 min**	2.13	-
	**190 min**	1.07	-
	**210 min**	1.14	-
**[Glucose]**	**170 min**	1.67	-
	**335 min**	1.49	-
	**345 min**	1.29	-
**[Glutamate]**	**345 min**	1.09	-
**[BKN]**	**150 min**	2.52	-
	**170 min**	1.13	-
	**310 min**	1.42	+
	**325 min**	1.53	+
	**335 min**	1.73	+
	**355 min**	1.22	+
**[IL-6]**	**150 min**	2.57	-
	**170 min**	1.50	-
	**190 min**	1.55	-
	**210 min**	1.66	-
	**345 min**	1.10	-
	**355 min**	1.22	-
**[K** ^**+**^ **]**	**170 min**	1.77	+
	**190 min**	1.19	+
	**210 min**	1.00	+
	**335 min**	1.04	+

**[substance]** denotes concentration of the substance. HHB = deoxy-haemoglobin, OHB = oxyhaemoglobin, THb = total haemoglobin, BKN = Bradykinin, IL-6 = interleukin 6, K^+^ = potassium, PEG = repetitive low-force exercise performed unilaterally on a pegboard, STR = STROOP test.

VIP values and sign of coefficient (coeff) are given. Note that the variables are grouped and sorted within each variable with respect to time point. A positive coefficient indicates that this variable will be associated with high PPT values and vice versa. Note the inverse construction of blood flow variables.

It was possible *in CON* to regress PPT of trapezius using baseline data (R^2^ = 0.66): Blood flow: VIP = 1.38 (+); [PINP]: VIP = 1.38(−); OHb: VIP = 1.36(−); [K^+^]: VIP = 1.35(+); [BKN]: VIP = 1.33(+); [5-HT]: VIP = 1.11(−); and THb: VIP = 1.10(−).

We also regressed PPT of trapezius using all time points (R^2^ = 0.51) (Table 5); blood flow at nine time points and [K^+^] at six time points were the most important regressors.

**Table 5:** **Regression of pressure pain threshold of trapezius in CON using the intramuscular variables (R** ^**2**^ **= 0.39)**

***Variable***	***Timepoint***	***VIP***	***Sign of coeff***
**Blood flow**	**150 min**	1.73	+
	**170 min**	1.90	+
	**190 min**	1.94	+
	**210 min**	2.40	+
	**310 min**	1.45	+
	**325 min**	1.62	+
	**335 min**	1.59	+
	**345 min**	1.95	+
	**355 min**	1.16	+
**OHB**	**21-40 min**	1.28	-
	**PEG-0-20 min**	1.67	-
	**recov1**	1.20	+
**THB**	**PEG-0-20 min**	1.38	-
	**21-40 min**	1.12	+
	**recov1**	1.29	+
**[Lactate]**	**190 min**	1.17	+
	**210 min**	1.37	+
**[Glucose]**	**310 min**	1.43	+
	**345 min**	1.04	+
**[Glutamate]**	**170 min**	1.54	+
**[BKN]**	**170 min**	1.12	+
**[IL-6]**	**210 min**	1.50	+
	**310 min**	1.42	+
	**325 min**	1.06	+
	**355 min**	1.17	+
**[K** ^**+**^ **]**	**150 min**	1.73	+
	**210 min**	1.71	+
	**325 min**	2.21	+
	**335 min**	1.35	+
	**345 min**	1.52	+
	**355 min**	1.57	+
**[5-HT]**		1.45	-
**[PINP]**		1.39	-

**[substance]** denotes concentration of the substance. OHB = oxyhaemoglobin, THb = total haemoglobin, BKN = Bradykinin, IL-6 = interleukin 6, K^+^ = potassium, 5-HT = serotonin, PINP = N-terminal propeptide of procollagen type I, PEG = repetitive low-force exercise performed unilaterally on a pegboard.

VIP values and sign of coefficient (coeff) are given. Note that the variables are grouped and sorted within each variable with respect to time point. A positive coefficient indicates that this variable will be associated with high PPT values and vice versa. Note the inverse construction of blood flow variables.

No significant regressions of PPT of tibialis anterior were found.

### Summing-up of multivariate analyses

A more diverse correlation pattern existed in TM compared with CON. A mix of factors comprised by blood flow related variables, algesic and metabolic substances were important for group membership (TM or CON). The three most important regressors of pain intensity at baseline in TM were positively correlated [5-HT] and [K^+^] and negatively THb. Prominent differences existed between CON and TM with respect to important regressors of PPTs of the trapezius muscles.

## Discussion

The major results of the present study were:

- [SP], but not the other investigated - mainly algesic - substances (i.e., 5-HT, IL-6, BKN, glutamate, LDH and PINP), was elevated in female workers with TM.
- The correlation pattern between algesics, metabolites and blood flow factors was more diverse in TM than in CON.
- Using a system-wide approach, increased [lacate], [pyruvate] and [K^+^] and decreased oxygenation characterized TM compared to CON; the classic algesic substances investigated had little importance.
- The pain intensity in TM correlated positively with [5-HT] and [K^+^] and negatively with oxygenation aspects.
- The most important regressors of PPT in TM were [BKN] (negatively), [IL-6] (negatively) and blood flow variables (positively).

### Algesics – traditional statistical analyses

In our recent study we reported significant group differences in [lacatate] and [pyruvate]. In the present study based on the same cohort we found increased [SP] among the primarily algesic substances examined [25]. SP is a mediator of neurogenic inflammation and associated hyperalgesia [48]. SP release in the periphery can occur as consequences of peripheral nociception and sensitization and SP binds to specific G protein-coupled tachykinin (NK) receptors [48]. Lymphocytes, granulocytes, and macrophages stimulated by SP produce inflammatory mediators as well as pro-inflammatory cytokines (IL-1, IL-6, and TNF) [48, 49], which in turn can stimulate adipocytes to synthesize SP [50]. Two MD studies of active trigger points of the trapezius in myofascial pain (MFP) found significant increases in [SP] [51, 52]. The present results agree with these two studies. However, the number of subjects in CON as well as the number of subjects in the studies of Shah et al. was low. The former circumstance may explain why [SP] was not an important regressor in the multivariate analyses (Tables 3, 4 and 5). Hence, the role of SP in chronic trapezius myalgia has to be investigated in larger cohorts.

We found no significant group differences in [glutamate], [IL-6], [BKN] and [5-HT]. Hence, it was not possible to confirm earlier studies of more severe TM [18–24, 53]. The lack of group differences for these algesics may be accounted by the degree of pain severity. Another possibility concerns the statistical methods used. Classical statistical methods can quantify the level of individual substances and assume variable independence when interpreting the results [27, 28]. There is a risk that more subtle alterations in several substances simultaneously are not detected using classical statistical methods [27, 28]. Only focusing upon a few substances in an explanatory phase of understanding peripheral alterations in chronic myalgia may not be fully comprehensive. Thus, taking into account system-wide aspects using multivariate analyses (Tables 3, 4 and 5) showed that peripheral alterations in algesics and metabolites together with blood flow aspects were linked to aspects of pain such as group membership, mechanical pain sensitivity and pain intensity.

### Different multivariate correlation patterns in CON and TM

The separate PCAs suggested a more diverse situation, as indicated by number of components, in TM than in CON. One reason for this may be that nociception and peripheral sensitization were present in TM and contributes to the increased degree of complexity. Another factor may be heightened myogenic activity (i.e., increased satellite cells and myonuclear content), which recently have been reported in the same subjects [54]. Also a recent proteomic study of the interstitium of chronic myalgia showed profound alterations compared to controls [55]. The sample size was higher in TM than in CON, increasing the chance of obtaining more components. However, the same pattern with more principal components in chronic TM than in controls was found in another project from our group with an equal number of subjects in both groups (unpublished analyses based on [20, 24, 56]).

### Regression of group membership

The regression of *group membership* showed that a mix of factors comprised of blood flow related variables, interstitial concentrations of metabolic, and, to a lesser extent, algesic substances were important. Low oxygenation (i.e., low blood flow and low OHb) and mainly high [lactate], [pyruvate], and [K^+^] characterized belonging to TM.

Originally, accumulation of lactate was thought to be an end product of anaerobic glycolyisis [57]. This interpretation is supported by trapezius muscle biopsy analyses from the same subjects of mRNA and proteins showing that the capacity of carbohydrate oxidation was reduced in TM compared with CON [58]. Such an inverse relationship between the metabolites and blood flow was observed in p1 of the PCA in CON, but no such relation existed according to the PCA of TM. As [lactate], [pyruvate] and blood flow variables were not correlated in TM they are not necessarily directly linked. In the majority of MD studies of chronic trapezius myalgia (and independently of degree of severity of the chronic myalgia) significant increases in [lactate] and [pyruvate] have been reported [18–20, 25, 53], but the results with respect to blood flow alterations have not been consistent [19, 20, 25]. Lactate is also produced during adequate oxygen provision [59]. Muscle [lactate] increases with exercise intensity [60]. Other possible explanations for increases are signs of mithochondrial insufficiency, physical inactivity due to pain [61, 62], and changes in the lactate-pyruvate metabolism via lactate dehydrogenase isoforms [59]. Lactic acid is dissociated at body pH [63]. Inflamed as well as ischemic tissues show lowered pH [64, 65]. Within the muscle cell, the protons can be buffered or released to the interstitium; to what extent buffering occurs in the interstitium is unknown [66]. Lactate together with adenosine triphosphate (ATP) facilitate the response of acid-sensing ion channel 3 (ASIC-3) to low pH [67–69]. ASIC channels are considered molecular transducers for nociception and mechanosensation. Other possible receptors for low pH are TRPV1 and 4, TRPC4 and 5 [64, 65, 70]. Lactate exposure can lead to reactive oxygen species (ROS) generation [71–73]. Hence, another possibility is that the increased [lactate] induced ROS, which may directly activate nociceptive pathways or activate algesics [74]. However, it has been suggested that pyruvate is an endogenous antioxidant that protects various tissues from ROS, cytokines, and ischemia/re-perfusion-induced injury [75–77].

Both the multivariate analysis at baseline and the analysis using all data identified [K^+^] (Table 3) as a significant regressor of group membership. This is in line with our earlier results in severe chronic myalgia where we have reported increased [K^+^] [56], although this was not found in our recent paper of the present subjects [25]. Increased activity and altered activity pattern of the chronic myalgic muscle have been reported and has also been confirmed for the present workers with TM [25, 78]. Potassium efflux via K^+^-channels and possibly increased metabolism, structural damage of cells, and depolarization-associated flows would also cause an interstitial potassium accumulation [56, 79]. It is not possible, based on this analysis of group membership, to determine whether the significantly increased [K^+^] in TM is directly involved in the nociception and perception of pain or if it is secondary consequences of pain e.g., deconditioning of the painful trapezius (see also below). Both pro-inflammatory and anti-inflammatory roles have been reported for IL-6 [80–82]. IL-6 also has metabolic properties and increases as a consequence of exercise can occur [81, 83, 84]. The extended analysis of group membership (Table 3) showed that [IL-6] at two time points were significant and lower in TM. Interestingly, [IL-6] tended to be higher at recovery and at baseline after PEG in CON than in TM (Figure 1), which may indicate an effect of exercise [83, 84]. Thus, a protective response may be lacking for TM. Possibly the lack of such increases in TM might be due to other muscle processes in TM than in CON.

[BKN] was only important at one time point (at 150 min) according to the comprehensive analysis of group (Table 3) and TM was associated with low [BKN] (Figure 1). This result concerning a classical algesic might be related to other muscle alterations e.g., in blood flow [85–87].

### Regression of pain intensity

As mentioned in the introduction ongoing pain is a common characteristic and core symptom in TM. Thus, the *pain intensity* variables reflect this habitual situation and are important to investigate with respect to the biochemical substances. The within-group regressions of pain intensity in TM showed that algesic substances were important as regressors; high pain intensity was positively associated with [5-HT] and [K^+^] and negatively with oxygenation factors (i.e., THb, HHb, and OHb) and [IL-6]. This result indicates that [K^+^] in fact has a role in chronic nociceptive processes as speculated above. In severe chronic trapezius myalgia was also found a positive correlation between [K^+^] and pain intensity [56]. In acute tissue trauma K^+^ is a component of the “inflammatory soup” and characterized as an algesic substance [88].

The fact that [5-HT] was important for pain intensity in TM agrees with its role as an algesic in the periphery as described in earlier studies of chronic myalgia [53]. In the periphery, 5-HT sensitizes afferent nerve fibres [89].

The negative correlation of [IL-6] with pain intensity in TM favours an anti-inflammatory effect of IL-6 as mentioned above [82]. The inverse relationship between pain intensity and the three Hb variables indicate insufficient oxygenation as a factor involved in the habitual pain intensity.

### Regression of mechanical pain sensitivity

When clinically examining the patient with neck-shoulder pain a frequently used sign is palpation of muscle tenderness; TM patients show increased tenderness over the trapezius. PPT measurements (i.e., mechanical pain sensitivity) is a more standardized way of determining muscle tenderness and concern the recognition of a new nociceptive stimulus (pressure) in painful or non-painful tissues. The fact that it was not possible to significantly regress *PPTs* of tibialis anterior reasonably indicates that the data registered from trapezius really represent local alterations in the trapezius. The regressions of PPT in the groups separately - both analyses of baseline data and all time points (Tables 4 and 5) - indicated that mechanical pain sensitivity in chronic pain conditions are not just consequences of levels of certain biochemical substances since several of the same and significant variables in both regressions had different signs: blood flow, [lactate], [IL-6], and [BKN]. Possible explanations for these results are that nociceptive, hyperalgesic, myogenic, and other processes are activated in TM but not in CON as discussed above [54, 55]. The separate analyses of mechanical pain sensitivity in the two groups of subjects give the opportunity to detect whether a substance is linked to presence of peripheral sensitization in TM. In TM, high concentrations of the two algesics BKN and IL-6 were significantly associated with low PPT. The results concerning BKN agree with reports that BKN is an algesic kinin with pro-inflammatory and hyperalgesic properties [5, 13, 90, 91]. No significant group difference in [BKN] was found in the present study or two other studies of TM [19, 24], but two small studies reported increased [BKN] in active trigger points in MFP [51, 52]. Increases in [BKN] might be important only in acute nociception or localized to the most painful areas of the afflicted muscle, hypotheses that future studies should address.

Our results also confirm that IL-6 has hyperalgesic properties with respect to recognizing a new nociceptive stimuli (i.e., mechanical pain sensitivity; PPT) in the chronically myalgic tissue [80, 81]. In agreement with the literature, we have reported both pro-inflammatory (with respect to mechanical pain sensitivity; PPT) and anti- inflammatory (with respect to pain intensity) roles for IL-6 [80–82]. The role of involvement of IL-6 and other cytokines in different aspects of chronic myalgia needs further investigation. The result that blood flow correlated positively with PPT in TM may be a consequence of e.g., alterations in algesics such as BKN and IL-6 [85–87].

### Algesics versus metabolites

The present study of active female workers with pain increased the possibilities to understand if muscle pain is maintained by metabolic and algesic substances and blood flow variables. Metabolites, potassium, and oxygenation factors, when considering the system-wide aspects, were the most important factors for group belonging. According to the traditionally statistical analyses, the classic algesic substances, except SP, were not increased, which is in contrast to other studies of chronic trapezius myalgia. There are several possible reasons for this observation: the workers with pain in the present study reported relatively low severity of their myalgia, metabolic alterations may precede detectable alterations in levels of algesics, or peripheral sensitization and other muscle alterations present in TM. The within-group analyses of TM gave some support to the latter explanation as classical algesics were significant regressors in the regressions of PPT and pain intensity and the multivariate correlation analyses (PCA) indicated a diverse pattern from controls.

### Strengths and limitations

As evident from above there exist several MD studies of human chronic myalgia. Hence, the feasibility of MD studies for the investigating biochemical alterations in humans are very good with respect to the participating patients and controls. With this said it is also important to recognize that our study has several limitations that have to be considered in future studies. It has not been possible to investigate the development over time for several of the biochemical substances (PINP, 5-HT and SP) due to lack of dialysate. A possible way to increase the number of substances investigated over time in the small volumes of dialysate is to use more sensitive techniques e.g. capillary electrophoresis and capillary electrochromatography [52]. In the present study a number of predetermined biochemical substances were analysed. However, our approach in the present study is to a certain extent explorative. Therefore more open-ended explorative methods could be used e.g. proteomics and metabolomics to gain further insights in the activated mechanisms and based on such analyses determine substances for detailed analyses [92, 93].

## Conclusion

With respect to the three aims of the study it can be concluded that 1) Only SP, but not the other investigated algesic substances, was elevated in female workers with TM; 2) Increased [lacate], [pyruvate] and [K^+^] and decreased oxygenation characterized TM compared to CON according to the multivariate analyses; the algesic substances had little importance; 3) In TM several of the algesics were of importance for the levels of pain intensity (5-HT and K+) and mechanical pain sensitivity (BKN and IL-6).

The present and earlier MD studies report muscle alterations in chronic myalgia. This may be an indication of peripheral contribution to maintenance of central nociceptive and pain mechanisms. It seems important to investigate if clinically effective treatments normalize these peripheral alterations in order to improve existing and develop new treatments for chronic myalgia.

## Endnote

^a^Not possible to perform in CON due the fact that these subjects had no pain.

## Electronic supplementary material

Additional file 1:**Data (all subjects taken together; Mean ± 1SD) at the different time points of the experiment presented in the earlier article [**[25]**].** For details, concerning comparisons with respect to group and time see the previous article [25]. This data together with data presented in *Supplement table b* were only used in the present multivariate analyses. PPT = pressure pain threshold, PEG = repetitive low-force exercise performed unilaterally on a pegboard, STR = STROOP test. (DOCX 14 KB)

Additional file 2:**Data (all subjects taken together; Mean ± 1SD) concerning Hb at the different time points of the experiment presented in the earlier article [**[25]**].** For details concerning comparisons with respect to group and time see the previous article [25]. This data together with data presented in *Supplement table a* were only used in the present multivariate analyses. OHB = oxyhaemoglobin, HHB = deoxy-haemoglobin, THb = total haemoglobin. PEG = repetitive low-force exercise performed unilaterally on a pegboard, STR = STROOP test. (DOCX 13 KB)

Below are the links to the authors’ original submitted files for images.

Authors’ original file for figure 1

Authors’ original file for figure 2
