# Supplementary material for: Algogenic substances and metabolic status in work-related Trapezius Myalgia: a multivariate explorative study
Source: BMC Musculoskelet Disord. 2014 Oct 28;15:357. doi: 10.1186/1471-2474-15-357 (PMC4223843; doi:10.1186/1471-2474-15-357)
Supplement: Supplementary file 1 — Additional file 1:Data (all subjects taken together; Mean ± 1SD) at the different time points of the experiment presented in the earlier article [[25]]. For details, concerning comparisons with respect to group and time see the previous article [25]. This data together with data presented in Supplement table b were only used in the present multivariate analyses. PPT = pressure pain threshold, PEG = repetitive low-force exercise performed unilaterally on a pegboard, STR = STROOP test. (DOCX 14 KB) [file 12891_2014_2294_MOESM1_ESM.docx]

| **Time points** | **Baseline** | **PEG** | **PEG** | **Recov** | **Baseline** | **STR** | **Recov1** | **Recov2** | **Recov3** |
| --- | --- | --- | --- | --- | --- | --- | --- | --- | --- |
| *Variables* | **150 min** | **170 min** | **190 min** | **210 min** | **310 min** | **325 min** | **335 min** | **345 min** | **355 min** |
| **Pain intensity (VAS; mm)** *(Mean(±SD))* | 22.9 (±23.2) | 28.6 (±25.2) | 37.1 (±29.8) | 29.9 (±28.1) | 26.5 (±27.2) | 29.0 (±28.1) | 27.6 (±27.5) | 25.7 (±27.2) | 26.7 (±27.7) |
| **Lactate (mmol/l)** *(Mean(±SD))* | 3.0  (±1.5) | 3.4  (±1.7) | 3.3  (±1.6) | 3.4  (±1.9) | 3.2  (±*2.2*) | 2.8  (±1.8) | 3.1  (±2.1) | 2.9  (±2.0) | 3.0  (±2.4) |
| **Pyruvate (µmol/l)** *(Mean(±SD))* | 112.0 (±64.8) | 134.8 (±75.8) | 136.6 (±77.8) | 136.9 (±93.2) | 138.6 (±96.3) | 122.4 (±76.0) | 135.2 (±78.1) | 127.7 (±81.9) | 140.6 (±118.9) |
| **Glucose (mmol/l)** *(Mean(±SD))* | 4.4  (±1.4) | 4.8  (±1.5) | 4.6  (±1.3) | 4.2  (±1.7) | 4.1  (±1.1) | 4.2  (±1.8) | 4.3  (±1.6) | 4.2  (±1.6) | 4.2  (±1.5) |
| **Potassium (mmol/l)** *(Mean(±SD))* | 3.9  (±0.3) | 4.1  (±0.2) | 4.1  (±0.2) | 4.0  (±0.2) | 3.9  (±0.1) | 3.9  (±0.1) | 3.9  (±0.1) | 3.8  (±0.1) | 3.8  (±0.1) |
| **Blood flow*** *(Mean(±SD))* | 0.54 (±0.12) | 0.48 (±0.14) | 0.48 (±0.13) | 0.53 (±0.13) | 0.50 (±0.14) | 0.51 (±0.15) | 0.51 (±0.14) | 0.50 (±0.13) | 0.49 (±0.14) |
| **PPT trapezius right** *(Mean(±SD))* | 340.0 (±133.0) | na | na | na | na | na | na | na | na |
| **PPT trapezius left** *(Mean(±SD))* | 316.4 (±131.4) | na | na | na | na | na | na | na | na |
| **PPT tibialis anterior** *(Mean(±SD))* | 364.0 (±181.8) | na | na | na | na | na | na | na | na |

*Arbitrary Unit, na = not applicable
