# Supplementary material for: Algogenic substances and metabolic status in work-related Trapezius Myalgia: a multivariate explorative study
Source: BMC Musculoskelet Disord. 2014 Oct 28;15:357. doi: 10.1186/1471-2474-15-357 (PMC4223843; doi:10.1186/1471-2474-15-357)
Supplement: Supplementary file 2 — Additional file 2:Data (all subjects taken together; Mean ± 1SD) concerning Hb at the different time points of the experiment presented in the earlier article [[25]]. For details concerning comparisons with respect to group and time see the previous article [25]. This data together with data presented in Supplement table a were only used in the present multivariate analyses. OHB = oxyhaemoglobin, HHB = deoxy-haemoglobin, THb = total haemoglobin. PEG = repetitive low-force exercise performed unilaterally on a pegboard, STR = STROOP test. (DOCX 13 KB) [file 12891_2014_2294_MOESM2_ESM.docx]

| **Time points** | **Baseline** | **PEG** | **Recov1** | **Recov2** | **STR** | **Recov** |
| --- | --- | --- | --- | --- | --- | --- |
| *Variables* |  |  |  |  |  |  |
| **OHb(µM)** *(Mean(±SD))* | -0.33 (±0.43) | -0.28 (±0.68) | 0.05  (±0.22) | 0.07  (±0.23) | 0.15  (±0.14) | 0.02  (±0.17) |
| **HHb (µM)** *(Mean(±SD))* | 0.01  (±0.07) | 0.01  (±0.09) | 0.01  (±0.09) | -0.01 (±0.10) | -0.01 (±0.06) | 0.01  (±0.09) |
| **THb (µM)** *(Mean(±SD))* | -0.07 (±0.14) | -0.06 (±0.20) | 0.06  (±0.25) | 0.06  (±0.26) | 0.14  (±0.19) | 0.03  (±0.23) |
