# Supplementary figures and images for: Algogenic substances and metabolic status in work-related Trapezius Myalgia: a multivariate explorative study
Source: BMC Musculoskelet Disord. 2014 Oct 28;15:357. doi: 10.1186/1471-2474-15-357 (PMC4223843; doi:10.1186/1471-2474-15-357)

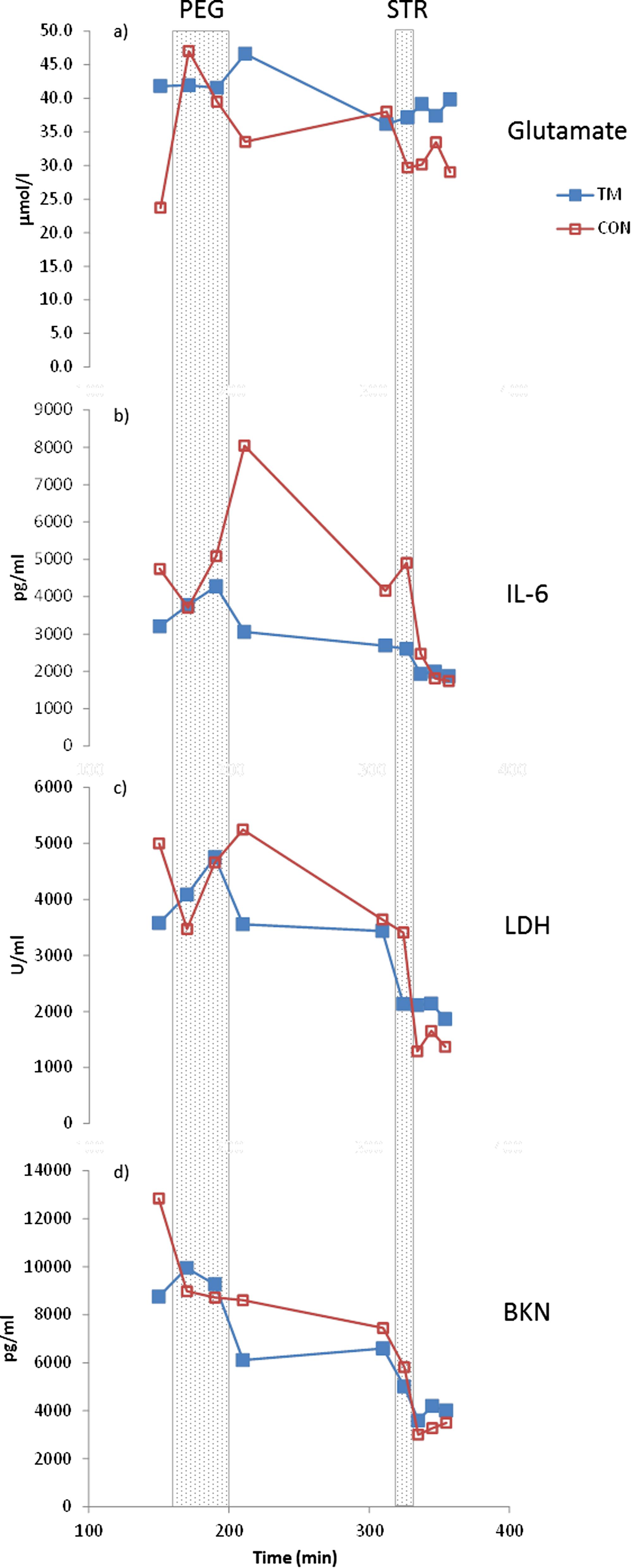

Supplement: Supplementary file 3 — Authors’ original file for figure 1 [file 12891_2014_2294_MOESM3_ESM.tif]

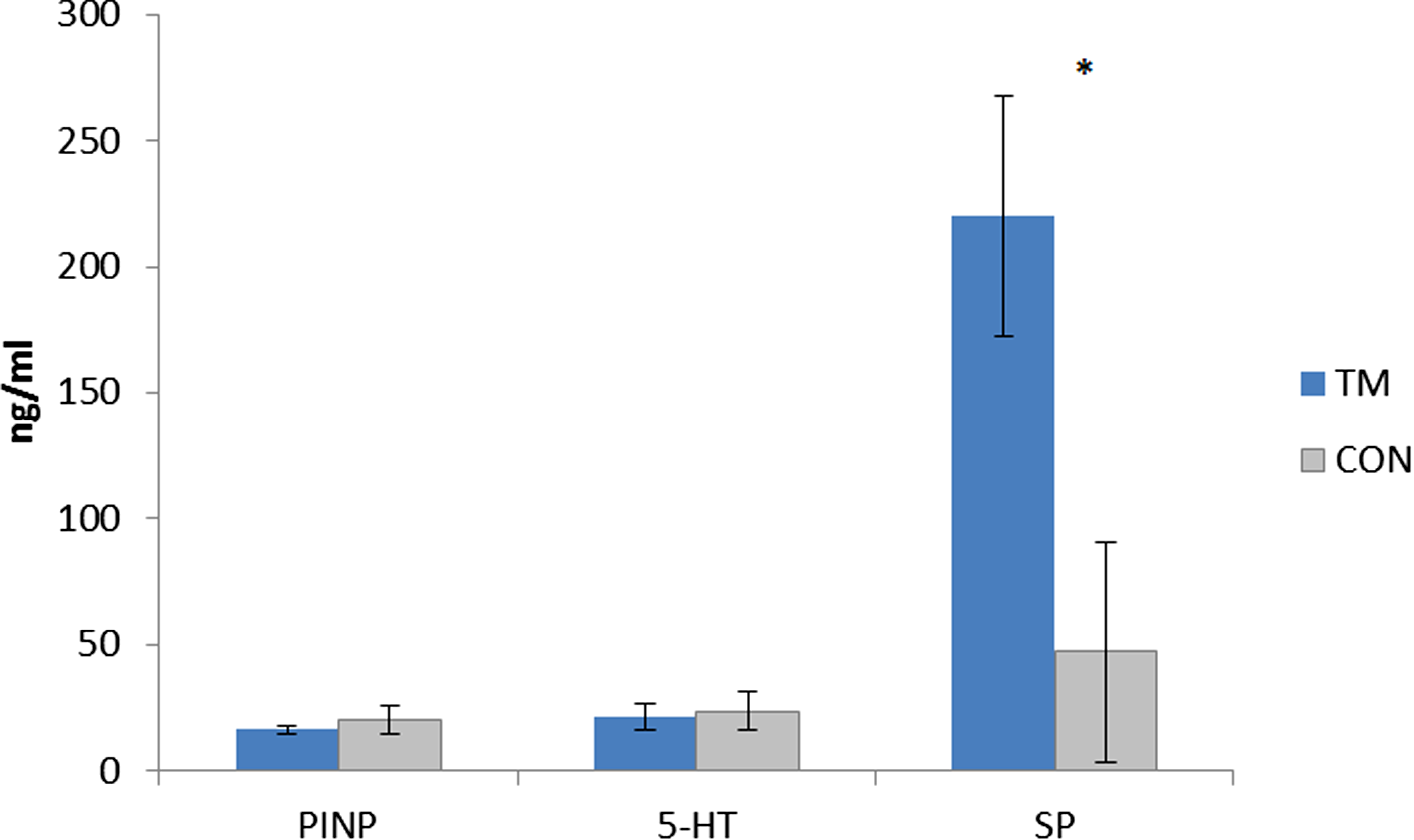

Supplement: Supplementary file 4 — Authors’ original file for figure 2 [file 12891_2014_2294_MOESM4_ESM.tif]
